# Epstein-Barr Virus in Systemic Lupus Erythematosus, Rheumatoid Arthritis and Multiple Sclerosis—Association and Causation

**DOI:** 10.3390/v4123701

**Published:** 2012-12-13

**Authors:** Andreas Lossius, Jorunn N. Johansen, Øivind Torkildsen, Frode Vartdal, Trygve Holmøy

**Affiliations:** 1 Institute of Immunology, Oslo University Hospital Rikshospitalet, Oslo, Norway; 2 Norwegian Multiple Sclerosis Competence Centre, Department of Neurology, Haukeland University Hospital, Bergen, Norway; 3 Department of Neurology, Akershus University Hospital, Lørenskog, Norway; 4 Faculty of Medicine, University of Oslo, Oslo, Norway

**Keywords:** systemic lupus erythematosus, rheumatoid arthritis, multiple sclerosis, Epstein-Barr virus, autoimmunity, B-cells, T-cells

## Abstract

Epidemiological data suggest that the Epstein-Barr virus (EBV) is associated with several autoimmune diseases, such as systemic lupus erythematosus, rheumatoid arthritis and multiple sclerosis. However, it is not clear whether EBV plays a role in the pathogenesis of these diseases, and if so, by which mechanisms the virus may contribute. In this review, we discuss possible viral and immunological mechanisms that might explain associations between EBV and autoimmune diseases and whether these associations represent causes or effects of inflammation and autoimmunity.

## 1. Introduction

Autoimmune diseases are believed to result from interactions between genetic, environmental and stochastic factors. Systemic lupus erythematosus (SLE), rheumatoid arthritis (RA) and multiple sclerosis (MS) are associated with certain HLA genes and more weakly to several other immune related genes [1]. However, most monozygotic twins are discordant [2], which leaves a large proportion of disease risk to be explained by factors other than heritability. Several observations argue for an important role of the environment in these diseases, including the influence of disease risk by month of birth [3], differences in worldwide geographical distribution [4] and, for MS, that people migrating before early adulthood adopt the disease risk of the country to which they migrate [5].

Several infectious agents have been suggested as environmental triggers of autoimmune diseases, but follow-up of initial studies has mostly failed to show that these agents play a specific role in the disease mechanisms. In MS, the list of proposed infectious causes is long and includes microbes, such as *Spherula insularis*, which was detected by microscopy of the cerebrospinal fluid (CSF) [6], but later shown not to exist [7]. Other candidates, such as paramyxovirus SV5, were suspected, based on immunological observations [8], which were later shown to be non-specific [9].

Epstein-Barr virus (EBV) was suggested early as an environmental trigger of autoimmune disease and remains a main candidate. Several studies have suggested that EBV is associated with autoimmune diseases, such as SLE, RA, MS, autoimmune thyroiditis, inflammatory bowel diseases, insulin-dependent diabetes mellitus, Sjögren’s syndrome, systemic sclerosis, myasthenia gravis and autoimmune liver diseases. A discussion of relevant mechanisms should include more than one disease. In this review, we focus on SLE, RA and MS. We first summarize evidence of association with EBV and then critically review viral and immunological mechanisms that might explain causation or merely association.

## 2. Evidence of Association

### 2.1 Serological Data

A possible association between EBV seropositivity and autoimmune diseases was first observed by coincidence in 1968 in a Brazilian population [10]. Since then, a multitude of studies have explored humoral immunity against EBV in SLE, RA and MS. North Americans of different ethnicities with SLE had an increased seroprevalence of EBV [11,12], and studies in other populations have shown an increased frequency of antibodies against EBV early antigens [13,14,15]. In one study, 99% of young SLE patients were seropositive for EBV compared to 70% of age-matched controls [16].

Almost all adult MS patients are seropositive for EBV, compared to 90% of healthy adults [17]. As for SLE, the differences in seroprevalence are more pronounced in lower age groups, where the general seroprevalence is lower [18]. Further, it has been demonstrated that MS risk is very low in individuals not infected with EBV, but increases sharply after EBV infection [19]. In a recent meta-analysis, previous EBV infection was actually found to be present in 100% of MS patients in studies using two independent methods of antibody detection [20]. The authors claimed that findings of MS patients without earlier EBV infection could be due to low sensitivity in the assays used for detection of antibodies.

In SLE and MS, titers of antibodies against EBV antigens are elevated compared to healthy controls [13,21,22,23], and for both diseases, this elevation seems to predate the first symptoms [24,25,26,27]. Anti-EBV nuclear antigen (EBNA)-1 IgG titers may also predict conversion from clinically isolated syndrome (CIS) to MS [28] and be a marker of MS disease activity [29], although the latter has been questioned by a recent study [30]. One study also indicated that exacerbations in MS were associated with reactivation of latent EBV infection [31], but these results have not been replicated [32,33]. For SLE, there is some evidence that titers of IgA antibodies against viral capsid antigen (VCA) may be associated with disease flares [34]. There is also some evidence that patients with RA have elevated serum titers of antibodies against EBV antigens [15,23,35,36,37]. However, in contrast to SLE and MS, it does not seem to be any association between the titers of antibodies and risk of subsequent RA [38]. The EBV seroprevalence rate among RA patients has been found to be the same as in healthy subjects [36,39].

In SLE, MS and RA, the humoral immune response against EBNA-1 may also be qualitatively different compared to healthy individuals. In MS, the strongest disease association has been demonstrated to a C-terminal EBNA-1 domain comprising amino acids (aa) 385–420 [40,41]. In a recent study including nine MS-discordant monozygotic twins, this was further narrowed down to aa 401–411, revealing increased response against this epitope in affected compared to healthy co-twins [42]. Lünemann and colleagues have demonstrated that children with MS have a broader IgG response against all three domains of the EBNA-1 protein and that some of the responses against the glycine-alanine repeat domain (aa 88–323) are directed against epitopes not found in the sera of demographically matched healthy peers [43]. A broadened EBNA-1 response with specific targets within the glycine-alanine repeat domain has also been demonstrated in pediatric SLE patients [12]. Antibodies reactive against an increased number of epitopes within the C-terminal part of EBNA-1 have been found in sera from adults with SLE and RA, supporting a broadened response towards EBNA-1 [44].

In MS and RA, it is also possible to measure the concentration of EBV-specific antibodies in body fluids that are contiguous with the diseased organs and, thus, reflect local synthesis of antibodies. Early studies on synovial fluid from RA joints did not show any evidence of the local production of antibodies against EBV [45,46,47]. Results are conflicting regarding intrathecal production of EBV specific antibodies in MS. One study using a large-scale protein expression clone array combined with epitope mapping identified the EBV antigens EBNA-1 and BRRF2 as frequent targets of the intrathecal antibody response in MS patients [48]. The authors also demonstrated that at least in some patients, the antibodies in the major oligoclonal bands specifically bound both EBV proteins. Another study showed a moderate, but significant increase of anti-EBNA-1 and anti-VCA antibody index (AI) in the CSF of patients with early MS [49], while the cytomegalovirus (CMV) AI was not elevated. However, recently, Otto *et al.* have demonstrated in a cohort of MS patients with EBV AI ≥2 that the intrathecal fraction of anti-EBV antibodies is low and does not differ from anti-measles antibodies [50]. This suggests that the local production of antibodies against EBV, like that against paramyxovirus SV5 [9], may be part of the polyspecific intrathecal immune response seen in this disease.

### 2.2 Infectious Mononucleosis

In developed countries, primary EBV infection may be delayed up to adolescence, in which case it presents as infectious mononucleosis (IM) in about 35%–50% [51]. Interestingly, a history of IM has been shown to be an independent risk factor for developing MS, increasing the risk about two times [52]. In contrast, this has not been demonstrated for SLE [53,54,55] or RA [56].

### 2.3 Cellular Immunity

Evidence of an aberrant T-cell response against EBV has been reported in SLE, RA and MS. An early study in SLE demonstrated that T-cells were unable to control the production of immunoglobulins (Ig) from EBV-infected B-cells [57]. Later studies have reported a functionally impaired EBV specific CD8+ T-cell response characterized by the decreased production of cytokines (interferon (IFN)-γ, tumor necrosis factor (TNF)-α, interleukin (IL)-2 and macrophage inflammatory protein-1β) and decreased cytotoxicity in SLE patients [58,59], which was not seen for CMV-specific CD8+ T-cells [59]. However, the frequencies of EBV specific CD8+ T-cells have in some studies been shown to be the same in SLE patients as in healthy individuals [58,60] and, in one study, slightly increased [59]. The frequency of IFN-γ secreting EBV-specific CD4+ T-cells has been reported to be increased [60].

The data are more conflicting in RA and MS. Early studies in RA suggested an impaired EBV specific T-cell response in blood. Thus, lymphocytes from RA patients underwent spontaneous transformation more rapidly and frequently than lymphocytes from healthy individuals [61], and T-cells were unable to control antibody production of EBV-infected B-cells [62]. Further, the frequency of EBV gp110-specific T-cells was shown to be lower in patients with RA [63]. Using A2/GLC or B8/RAK tetramers, another study demonstrated similar CD8+ T-cell frequencies against these lytic and immunodominant EBV epitopes in RA patients and healthy controls. In patients with RA, however, a lower fraction of these CD8+ T-cells produced IFN-γ in response to their peptide antigens [64]. In contrast, a more recent study has reported an increased frequency of CD8+ T-cells responding upon stimulation with pooled lytic and latent EBV antigens [37].

Also in MS, early *in vitro* studies suggested an impaired CD8+ T-cell control of EBV infected B-cells [65,66]. Supporting this, Pender and colleagues found lower frequencies of CD8+ T-cells reacting upon *in vitro* stimulation with EBV lymphoblastoid cell lines (EBV-LCL) [67]. However, still more studies have demonstrated increased EBV specific CD8+ T-cell responses in MS. Cepok and colleagues found an increased frequency of EBV-LCL reactive CD8+ T-cells in blood of MS patients compared to healthy donors [48], while Hollsberg and colleagues demonstrated an increased frequency of CD8+ T-cells responding to a lytic and a latent EBV epitope in blood of MS patients compared to healthy controls [68]. Finally, a large study including 91 individuals with demyelinating disease, demonstrated an increased frequency in blood of CD8+ T-cells responding to a peptide pool comprising 18 HLA class I restricted peptides of several lytic and latent proteins, compared to 28 patients with other neurological diseases and 20 healthy controls [69]. This study also demonstrated that the CD8+ T-cell response was inversely proportional to disease duration. Thus, patients with CIS displayed higher frequencies of EBV specific T-cells than patients with established MS, and this frequency decreased in 12 out of 13 CIS patients followed prospectively for one year [69]. This temporal evolution of the EBV specific CD8+ T-cell response in MS and CIS could possibly explain the discrepancies between the latter studies and the findings of Pender and colleagues [67]. The CD4+ T-cell response against the latent cycle antigen EBNA-1 has been shown to be selectively increased and exhibit a broadened specificity in patients with MS [70].

As for antibodies, it is also possible to study T-cells from body fluids contiguous with the diseased organs in RA and MS. EBV specific CD8+ T-cells were shown early to be enriched in the synovial fluid compared to blood in patients with RA [71,72]. However, subsequent studies revealed that EBV specific CD8+ T-cells and, in several cases, also CMV-specific CD8+ T-cells could be locally enriched in other chronic inflammatory joint disorders (Reiter’s syndrome, psoriatic arthritis, ankylosing spondylitis, osteoarthritis) and in a few patients with inflammatory processes affecting other organs (uveitis, encephalitis and MS) [73,74]. In contrast, another study showed that EBV-specific, but not CMV-specific, CD8+ T-cells were enriched in the CSF of patients with MS [49]. No such accumulation was observed in CSF from patients with other neuroinflammatory disorders [49]. We have found that CD4+ T-cells from the CSF of MS patients respond vigorously upon stimulation with EBV-transformed B-cells [75] and were later able to clone EBV DNA polymerase and EBNA-1 specific CD4+ T-cells from this compartment [76,77]. It is, however, not known whether the frequency of EBV-specific CD4+ T-cells differs between CSF and blood or in CSF between MS patients and controls.

### 2.4 EBV Viral Load and Expression of Viral Genes in Blood

SLE patients have on average a five-to-40-fold increased EBV genome load in blood compared to healthy individuals [59,60,78,79], and this is shown to be due to an increase in latently infected memory B-cells [80]. This increase seems to be independent of treatment with immunosuppressive agents [60,78,80]. The number of infected B-cells correlates with disease activity [80], and the viral load peaks after initiation of disease flares [59]. Patients with SLE also have an aberrant expression of the lytic gene BamHI Z leftward open reading frame-1 (BZLF-1) and the latent membrane protein (LMP)-1 and -2 genes in blood [80]. One study found that EBV viral loads correlated inversely with the frequency of EBV-specific CD4+ T-cells and positively with the frequency of EBV-specific CD8+ T-cells [60], but another study failed to replicate this [59].

Likewise, patients with RA have increased EBV DNA load in peripheral blood mononuclear cells (PBMC) [37,81,82,83]. The viral load was found to be the same in patients receiving or not receiving immunosuppressive treatment [83] and did not increase further after long-term treatment with methotrexate or TNF-α inhibitors [84]. Lünemann and colleagues have demonstrated in patients with RA that the number of EBV-specific CD8+ T-cells correlates positively with the viral load, whereas CD4+ T-cell responses against EBV and CD8+ T-cell responses to CMV antigens do not [37].

Conflicting results have been reported regarding EBV viral load in the blood of MS patients. Several studies have failed to show any difference in quantity of EBV nucleic acids between blood from patients and controls [70,85,86]. In contrast, one study has demonstrated an increased EBV DNA load in the PBMC of patients with CIS compared to healthy EBV carriers [28]. Others have found an increased incidence of EBV DNA in serum during exacerbations compared to stable disease periods [31]. Finally, a study has shown that the presence of plasma EBV DNA is associated with increased risk of MS [87].

### 2.5 Detection of Virus in Diseased Organs

RA joints and MS brains have been scrutinized for evidence of EBV infection for decades. In both diseases, conflicting results have been reported. Early studies on RA synovial membranes failed to detect EBV using indirect immunofluorescence [88] or hybridization with DNA probes for several viral genes [89]. Later studies using *in situ* hybridization searching for EBV encoded RNAs (EBERs) or BamHl H leftward open reading frame-1 either failed to detect these in synovial membranes [90] or could identify EBERs in 8 to 62% of the cases [82,91,92,93]. However, only one study included a control virus [94]. Notably, in this study, EBV was detected in 16.7% and CMV in 20.7% of cases. Other groups have used PCR on synovial membranes or synovial fluid, with EBV positive cases ranging from 6% to 47% [82,92,95,96,97]. However, one study has also reported EBV in synovial lymphocytes in 33% of patients with reactive arthritis [95].

Studies using *in situ* hybridization for viral RNA failed to detect any EBV transcripts in MS brains [98,99]. However, a later study detected EBV-infected B-cells and plasma cells in brains from 21 of 22 MS cases by *in situ* hybridization for EBERs and immunohistochemistry for LMP-1 and LMP-2A [100]. EBV infected B-cells were mainly found in follicle-like structures in the meninges and in active brain lesions. However, this study did not include a control virus, and the authors were not able to detect EBV DNA in the CSF of MS patients with real time PCR. Several other groups have not been able to confirm the presence of EBV in the CNS of MS patients. In these negative studies, various methods, including *in situ* hybridization for EBERs [101,102,103], immunohistochemistry [101,102,103,104] or PCR for EBV RNA [101,102,104,105] or EBV DNA [101,102,105], have been used. Part of the reason for these discrepant results could be that the number of B-cell infiltrates differed between the different studies [106]. However, several groups have also studied tissue from some of the tissue blocks used in the positive study [100], without detecting EBV infected cells [101,102]. Recently, one study replicated the findings of EBER+ B-cells in active MS lesions [107]. The authors did, however, also find evidence of EBER+ cells in two of two studied cases of stroke, suggesting that the phenomenon may not be MS-specific. The associations between EBV and SLE, RA and MS are summarized in Table 1.

**Table 1:** Summary of associations between EBV and SLE, RA and MS.

	SLE	RA	MS	References
Increased EBV seroprevalence	+	–	+	[11,12,13,14,15,16,17,18,19,20], [36], [39]
Elevated serum titers of anti-EBV antibodies	+	+	+	[14], [15], [21,22,23,24,25,26,27], [35,36,37]
Elevation of antibodies predates symptoms	+	–	+	[24,25,26,27], [38]
Association with infectious mononucleosis	–	–	+	[52,53,54,55,56]
Aberrant systemic T-cell response against EBV	+	+	+	[37], [48], [57,58,59,60,61,62,63,64,65,66,67,68,69,70]
Increased local T-cell response against EBV	N/A	+	+	[49], [71,72,73,74]
Increased EBV viral load in blood	+	+	+/–	[28], [31], [37], [58], [60], [70], [78,79,80,81,82,83], [85,86,87]
Virus detected in diseased organ	N/A	+/–	+/–	[81], [88,89,90,91,92,93,94,95,96,97,98,99,100,101,102,103,104,105], [107]

## 3. Viral mechanisms

### 3.1 Infection and Immortalization of Autoreactive B-cells

EBV infects B-cells through binding to the viral envelope glycoprotein 350 to the B-cell complement receptor 2, CD21 [108,109]. Infection of naive B-cells leads initially to the expression of nine virally encoded proteins (EBNA 1-6 and three LMPs), and this expression pattern is referred to as the latency III program [108]. Infected cells enter the germinal center (GC) in the tonsil and change their viral transcription program to express only EBNA-1, LMP-1 and LMP-2 (latency II). It is demonstrated *in vitro* that LMP-1 serves a signal that normally comes from the CD40 signal transduction pathway initiated by CD4+ T-cells [110,111], whereas LMP-2A mimics a constitutively activated B-cell receptor [112]. LMP-1 and LMP-2A assist infected naive B-cells in the GC process and help EBV to gain access to the memory B-cell pool, where it enters a truly latent state (latency 0/I).

It has been hypothesized that EBV might infect autoreactive naive B-cells, and drive these into latently infected memory B-cells resistant to apoptosis. These cells could then lodge in the organs, where their target antigens are expressed and act as antigen presenting cells (APC) rescuing autoreactive T-cells [113,114]. Based on *in vitro* studies, it was previously thought that LMP-1 and LMP-2A were sufficient to drive infected B-cells through the GC even in the absence of antigen [108] and that this mechanism could allow the escape of autoreactive B-cells. Indeed, in transgenic mouse models, LMP-2A expression in B-cells prevented induction of energy in autoreactive B-cells [115] and led to the bypass of tolerance checkpoints, resulting in high serum levels of autoantibodies and to the development of lupus-like disease [116]. However, a more recent *in vivo* study showed that EBV infected GC B-cells, while expressing LMP-1 and LMP-2A, retained both phenotypic and functional characteristics of normal GC B-cells [117]. The authors suggested a more modest role for these EBV latency II proteins, perhaps only supplementing physiological signals. In line with this idea, Tracy and colleagues did not find any evidence that EBV favored the survival of autoreactive B-cells during IM [118]. On the contrary, EBV infected memory B-cells were found to express lower levels of self- and poly-reactive antibodies than their uninfected counterparts. Further, antibodies made by EBV positive and EBV negative B-cell populations showed no difference in the distribution of isotypes, VH and JH usage, the extent of somatic hypermutations or CDR3 length.

### 3.2 EBV Infection of Other Cell Populations

In addition to B-cells, EBV may also target other cells *in vivo,* including T-cells [119,120] and NK cells [121]. It has been proposed that infection of T-cells could impair T-cell apoptosis through inactivation of NF-κB by the expression of the EBV ZEBRA protein [122] and enhanced p53 signaling [123]. Apoptosis of autoreactive T-cells is believed to be fundamental for the maintenance of self-tolerance, but it remains to be established whether EBV-mediated impairment through the proposed mechanism is relevant in the pathogenesis of autoimmune diseases.

EBV infection of human astrocytes and brain microvascular endothelial cell lines has been demonstrated *in vitro* [124,125]. It has been proposed that reactivation of latent EBV infection in brain endothelial cells could up-regulate cytokines, chemokines and adhesion molecules that could facilitate access of lymphocytes to the brain [125]. The relevance of this hypothesis, which rests on the unproven assumption that EBV infects human endothelial cells *in vivo,* remains to be shown. Notably, evidence of the latent or active EBV infection of endothelial cells has so far not been reported in MS brains [98,99,100,101,102,103,104,105,107].

### 3.3 Innate Immunity

It has been postulated that EBV could exacerbate inflammation in autoimmune diseases by enhancing innate immune responses [126]. Thus, LMP-1 has been shown *in vitro* to prime production of IFN-α in EBV-infected B-cells [127] and to upregulate the expression of the B-cell activating factor of the tumor necrosis family (BAFF) [110], IL-6 [128] and IL-10 [129].

Serum levels of IFN-α are increased in SLE and correlate with disease activity [130]. The mechanism behind this probably involves both Toll-like receptor (TLR)-dependent and independent mechanisms [131]. Plasmacytoid dendritic cells from healthy individuals have been shown to produce large amounts of IFN-α when cultured in the presence of EBV DNA and RNA, through a mechanism involving engagement of TLR-9 and -7 [132]. Others have found that LMP-1 and IFN-α are co-expressed in PBMC from SLE patients [133]. The authors of this study suggest that TLR-7-stimulation of EBV infected B-cells may induce LMP-1-mediated secretion of IFNs and, thereby, drive a vicious cycle, leading to enhanced antibody production and tissue damage.

In MS, Tzartos and colleagues detected overexpression of EBERs and IFN-α in active areas of white matter lesions, whereas neither IFN-α nor EBERs were expressed in inactive lesions [107]. The authors also demonstrated *in vitro* that EBERs are able to elicit IFN-α production in TLR-3-expressing HEK cells and suggested that EBV could drive inflammation through activation of innate immune responses. Serafini and colleagues observed a strong expression of BAFF in the cytoplasm of EBV infected B-cells in acute MS lesions and ectopic B-cell follicles, and they proposed that EBV activation signals might be amplified by the autocrine and paracrine actions of BAFF [134].

### 3.4 The State of EBV Infected B-cells in a Chronically Inflamed Environment

While EBV infection and reactivation possibly could trigger inflammation, it is also conceivable that the proinflammatory environment in autoimmune diseases may alter the regulation of EBV latency, leading to reactivation of the virus. Supporting this idea, it has been demonstrated *in vitro* that several cytokines, including IFN-α [135], IL-10 [136] and IL-21 [137], induce LMP-1 expression in EBV infected B-cells. Notably, it has been shown that the expression of these cytokines are increased in diseased organs of patients with MS [107,138,139] and RA [140,141,142,143] and in the blood of patients with SLE [130,144,145]. Moreover, cross-linking of the B-cell receptor of EBV infected B-cells activates transcription of BZLF-1, resulting in lytic EBV replication [146]. Likewise, activation of B-cells resulting in terminal differentiation into plasma cells seems to initiate the replicative EBV cycle *in vivo* [147]. Such mechanisms could potentially explain i) higher EBV viral loads in serum of patients with SLE and RA, ii) increased expression of LMP-1 and BZLF-1 in the blood of patients with SLE, iii) a possible detection of EBV in the inflamed organs of RA and MS and iv) a secondary increased humoral and cellular response against EBV (as detailed in section 2.4, section 2.5, section 2.1 and section 2.3).

There is a significant accumulation of memory B-cells in or near the diseased organs of patients with RA [148] and MS [149,150]. As EBV resides in latently infected memory B-cells, such accumulations increase the probability that EBV will be present and exposed to the local inflammatory environment. Ectopic lymphocyte aggregates and B-cell follicles have been detected in some patients with long-standing MS [151,152] and would be expected to harbor some EBV infected B-cells. They have indeed been reported to be major sites of EBV persistence [100], but this finding has so far not been replicated (detailed in section 2.5).

### 3.5 Transactivation of Human Endogenous Retroviruses

Human endogenous retroviruses (HERVs) have been proposed to link infection and autoimmunity [153]. Hence, EBV induces transcription of the endogenous retrovirus HERV-K18, which encodes a superantigen that activates the T-cells carrying the T-cell receptor (TCR) Vβ7 and Vβ13 families [154]. The expression of HERV-K18 has been shown to be elevated in peripheral blood and inflamed joints of patients with juvenile RA, but not in the peripheral blood of pediatric patients with SLE [155]. One of the three HERV-K18 Env alleles (K18.3) was reported to be associated with MS in a case-control study, although the replication analysis in an independent sample set was non-significant [156]. Interestingly, human herpesvirus-6A may also be associated with MS [157] and is, like EBV, also shown to transactivate HERV-K18 [156]. However, there is no evidence for the selective expansion of T-cells carrying the TCR Vβ7 and Vβ13 families in the brain [158], blood or CSF (Lossius *et al.*, unpublished) of MS patients, or in the synovial tissue from joints of RA patients [159,160].

Recently, EBV has also been demonstrated *in vitro* to activate HERV-W, also known as MS-associated retrovirus (MSRV), in astrocytes, B-cells and monocytes from MS patients [161]. MSRV has repeatedly been isolated from patients with MS [162,163,164] and has previously been shown *in vitro* to stimulate T-cells carrying TCRs of the Vβ16 family [165] and to induce the production of several cytokines [166,167,168].

## 4. Immunological Mechanisms

### 4.1 Molecular Mimicry and Mistaken Self

Molecular mimicry, first proposed by Fujinami and Oldstone [169], is one of the main hypotheses on how infections may cause autoimmunity. Sequence or structural similarities between microbial and self-antigens are believed to cause cross-reactivity of T-cells, B-cells and antibodies. It has been shown that cross-reactive antibodies are involved in the pathogenesis of Sydenham chorea, Guillain-Barré syndrome and HTLV-1 associated myelopathy. In contrast, although there is strong circumstantial evidence [170,171] and the mechanism is shown to be relevant in animal models of autoimmune diseases [172], cross-reactive T-cells have so far not been shown to mediate human disease.

In SLE, autoantibodies against epitopes on SmB’ and SmD1 have been shown to cross-react with different domains of EBNA-1 [173,174]. Rabbits immunized with the EBNA-1 motif PPPGRRP acquired lupus-like autoimmune disease [175]. Immunization of mice with the entire EBNA-1 protein led to the development of anti-dsDNA and anti-Sm antibodies [176]. Furthermore, antibodies against Ro (aa 169–180), the earliest detectable autoantibodies in a subgroup of SLE patients, have been shown to cross-react with EBNA-1 (aa 58–72). Immunization of rabbits with either peptide induced a humoral immune response against both antigens, with the subsequent epitope spreading to other antigenic determinants of Ro and to other lupus associated autoantigens. The rabbits eventually developed SLE-like symptoms, such as leukopenia, thrombocytopenia and renal dysfunction [177].

Anti-citrullinated protein antibodies (ACPA) are present in the sera of most patients with RA. These post-translational modified proteins are products of peptidyl arginine deiminase (PAD), the enzyme catalyzing the conversion of arginine residues into citrulline [178]. In the inflamed synovium, dying cells might leak PAD, which could become activated by the high extracellular calcium concentration. Alternatively, the enzyme could be activated through calcium influx in apoptotic cells. In such settings, EBV proteins may become substrates for post-translational citrullination and, thereby, possible targets for ACPA. Supporting this idea, a study detected antibodies specific for a citrullinated EBNA-1 peptide (aa 35–58) in approximately 50% of RA sera and in less than 5% of normal and disease control sera, and the authors speculated whether EBV infection may play a role in the induction of ACPA [179].

HLA molecules carrying the amino acid sequence QKRAA, QRRAA or RRRAA at positions 70–74 of the DRβ1 chain are associated with ACPA positive RA [180]. The QKRAA determinant is also expressed on the EBV protein gp110 and has been shown to be a target of humoral and cellular immune responses in humans [181]. One group found reduced frequencies of T-cells responsive to gp110 in patients with RA [63]. However, others have found an increased humoral and cellular response in RA against gp110, but also against several other microorganisms that express the QKRAA motif, including Brucellaovis and Lactobacillus lactis [182].

The HLA class II allele DRB1*1501 is the strongest genetic risk factor for MS. DRB1*1501 is in strong linkage disequilibrium with DRB5*0101, and these two alleles may be involved in molecular mimicry between EBV and the myelin basic protein (MBP) in MS. Thus, Lang and colleagues demonstrated that the Hy.2E11 TCR from an MS patient cross-recognized a DRB1*1501-restricted MBP peptide and a DRB5*0101 restricted EBV DNA polymerase peptide [183]. A later study, which used humanized mice carrying DRB1*1501 and DRB5*0101, as well as the cross-reactive Hy.2E11 TCR, suggested a functional epistasis between DRB1*1501 and DRB5*0101 [184]. Thus, DRB5*0101 ameliorated experimental autoimmune encephalomyelitis in mice also carrying DRB1*1501. The relevance of these observations for MS remains to be proven. We have found, however, that CD4+ T-cells cross-recognizing these particular MBP and EBV epitopes were prevalent in the CSF of an MS patient [75]. Others have shown that MS patients have clonal expansions of EBNA-1 specific T-cells in blood that recognize myelin antigens more frequently than other tested autoantigens [185]. These cross-reactive T-cells co-produced IFN-γ and IL-2, which is a characteristic of polyfunctional T-cells.

The small heat shock protein αB-crystallin is expressed in MS lesions, but not in normal white matter and has been identified as a candidate autoantigen in MS [186]. Human B-cells do not usually express αB-crystallin, but do so upon EBV infection in which case they also may present αB-crystallin peptides on their HLA class II alleles molecules to αB-crystallin specific T-cells [187]. This gave rise to the “mistaken self hypothesis” [188], suggesting that peripheral EBV infection of lymphoid cells prime the human T-cell repertoire not only to microbial antigens, but also to *de novo* expressed αB-crystallin in infected lymphoid cells. Further studies in transgenic mice have shown that αB-crystallin is a negative regulator of inflammation and apoptosis in the CNS [189]. Sequence similarities have been found *in silico* between αB-crystallin and EBNA-1 (aa 385–420) [190]. The levels of antibodies specific for this part of EBNA-1, the presence of HLA DRB1*15 and the absence of HLA A*02 have been shown to be interacting risk factors in MS, supporting a role for this EBNA-1 domain in the immunopathogenesis [40,41].

### 4.2 Bystander Activation and Epitope Spreading

Another theory on how infections may induce or augment autoimmunity is referred to as bystander activation. In this scenario, the inflammatory setting of an infection promotes activation of or expansion of previously activated, autoreactive lymphocytes [191,192,193]. Bystander activation may be antigen-independent, when autoreactive lymphocytes are stimulated by cytokines or superantigens, or antigen-dependent in the setting of tissue destruction and presentation of self-antigens by APC to autoreactive T- or B-cells [192]. The latter mechanism may be amplified by further tissue damage and presentation of additional self-antigens, resulting in activation of lymphocytes of other specificities. This process is known as epitope spreading and might involve new epitopes on the same [194] or on different molecules [195]. B-cell epitope spreading has been shown to take place in rabbits acquiring lupus-like disease after immunization with an EBNA-1 peptide (detailed in section 4.1) [177]. A possible broadened humoral response against EBNA-1 in MS [43], SLE [12,44] and RA [44] and the broadened T-cell response against this antigen in MS [70] might be a result of intramolecular epitope spreading.

One might speculate whether the severe systemic inflammation during IM may promote bystander activation and expansion of autoreactive lymphocytes, potentially explaining the association with MS. However, it remains to be proven whether such a mechanism could be relevant. Notably, a recent study found evidence of activation, but not expansion, of the influenza and CMV-specific memory T-cell pools during IM [196].

Bar-Or and colleagues have proposed that abnormal B-cell cytokine responses in patients with MS might mediate bystander activation of disease-relevant T-cells, resulting in increased disease activity [197]. Supporting this in MS, they demonstrated that depletion of B-cells *in vivo* and *ex vivo* reduced inflammatory CD4+ and CD8+ T-cell responses [197]. Soluble products from B-cells of untreated MS patients reconstituted the diminished T-cell responses, an effect that seemed to be partly mediated by lymphotoxin and TNF-α.

### 4.3 Dual and Chimeric TCRs

An αβ T-cell carries a TCR consisting of an α and β chain, in which the variable regions are products of V-J and V-D-J gene recombinations, respectively. However, studies in humans have revealed that about 30% of αβ T-cells express functional dual Vα TCRs and 1% expresses dual Vβ TCRs. In addition, there are some T-cells expressing different chimeric TCRs generated by a single Vα or Vβ combining with two different Vβ or Vα, respectively. A potential mechanism giving rise to autoimmunity could be that two different TCRs allow autoreactive T-cells to escape the negative selection mechanisms in the thymus [198]. It has recently been demonstrated that a viral infection in mice could trigger CNS autoimmunity by activating T-cells expressing different TCRs specific for viral and myelin antigens [199]. Such a mechanism has, however, so far not been linked to EBV.

### 4.4 Polyspecific B-cell Activation

There are some indications that raised serum titers of antibodies against EBV in autoimmune diseases could be caused by polyspecific B-cell activation. Memory B-cells have been shown to proliferate and differentiate into plasma cells in response to polyclonal stimuli, and this could represent a natural mechanism for the maintenance of lifelong serological immunity [200]. Such stimuli include bystander T-cell help through CD40L and bacterial CpG DNA stimulation of TLRs [200]. Notably, both these mechanisms have been shown to be dysregulated in autoimmune diseases [201,202,203,204,205,206]. Indeed, SLE is associated with hypergammaglobulinemia, and one recent study demonstrated elevated titers of antibodies against several microbes, including EBV [13]. Besides MS, intrathecal oligoclonal bands may also be found in several infectious diseases of the central nervous system, in which case the IgG bands are directed against the etiological agent [207,208]. In MS, there is an intrathecal synthesis of antibodies against several viruses [209], but these antibodies are not part of the major oligoclonal CSF IgG bands [210]. The intrathecal immune response against EBNA-1 in MS may be a part of this local polyspecific humoral response [50]. Supporting this, recombinant antibodies made from single sorted expanded plasma cells in the CSF of a patient with subacute sclerosing panencephalitis were specific for measles virus [211], the causative pathogen, whereas recombinant CSF antibodies from MS patients did not react with EBV [105]. The serum and CSF measles antibody levels in MS have been found to increase over time [212], further supporting the idea of a polyspecific humoral response. Moreover, one prospective study has found that elevated serum titers of IgG not only against EBNA-1, but also against herpesvirus-6 were associated with increased risk of MS [213]. A recent study demonstrated distinct profiles of antibodies against herpes viruses in neuromyelitis optica and MS [214]. As previously found, MS patients had elevated levels of antibodies against EBNA-1, but they also showed elevated levels of antibodies against varicella zoster virus compared to the NMO patients.

### 4.5 Accumulation of EBV-specific CD8+ T-cells in Sites of Inflammation

As detailed in section 2.3, EBV specific CD8+ T-cells are enriched in or near the diseased organs of patients with RA [71,72] and MS [49]. This could reflect a local immune response against EBV in the diseased organs, but other explanations have also been suggested. EBV-specific CD8+ T-cells have also been reported to accumulate in synovial fluid from patients with psoriatic arthritis, osteoarthritis and Reiter’s syndrome [73,74], indicating an unspecific “trapping” of virus-specific T-cells within inflamed sites. In such a scenario, frequent reactivations of the virus in the periphery activate new T-cells, which favor homing to inflamed tissues, due to increased sensitivity to chemotactic factors [73]. In line with this idea, acute lymphocytic choriomeningitis virus infection in mice harboring mycobacterium-induced granulomas led to substantial accumulation of virus-specific T-cells in the inflamed granulomas [215].

## 5. Conclusions

After decades of epidemiological, viral and immunological research, it is still an open question whether the observed association between EBV and autoimmunity represent causation. It is striking that for SLE, RA and MS, most associations with EBV are valid for more than one disease. For instance, both SLE and MS are associated with an increased seroprevalence of EBV; all diseases are associated with elevated serum titers of anti-EBV antibodies and a perturbed T-cell response against the virus. It seems likely that at least some of these shared associations reflect shared mechanisms. Such mechanisms could possibly involve B-cells, which are central players in the pathogenesis of SLE [216], RA [217] and MS [218] and also the main site of EBV persistence [219].

Basic research on possible mechanisms is hampered by the fact that EBV does not infect animals commonly used in models of autoimmune diseases. Several research groups have developed humanized mouse models of EBV infection [220,221], and a recent study demonstrated erosive arthritis resembling RA in the majority of such mice [222]. However, the mouse models are based upon transfer of human hematopoietic stem cells and are complicated with graft *versus* host disease [223], which is a major confounder when studying autoimmunity.

Future research should continue to investigate hypotheses on EBV as a causative risk factor in autoimmunity. Conversely, it is equally important to explore whether the associations with EBV could merely represent the effects of autoimmune inflammation. One important task in MS is to identify the mechanisms driving the increase in serum titers of anti-EBNA-1 antibodies prior to the onset of symptoms, as these may represent pathogenic events [27]. EBV vaccination programs could possibly give reliable, although late, answers to the question of cause and effect. On one hand, vaccination studies may not be justified for autoimmune diseases, given their rarity, the lack of definitive proof of causality, the possible risk of eliciting harmful immune responses against the vaccine and the risk of postponing primary infection to a more vulnerable age. On the other hand, vaccination might also prevent EBV-associated malignancies and IM, which in some cases shows a protracted course and even lead to serious complications in about 1% of the cases [224]. It is, of course, a prerequisite to develop efficient vaccines with a proven safety profile. To date, several vaccines are under development [224], one of which has shown some efficacy in a phase II trial in preventing infectious mononucleosis, but not in preventing asymptomatic infection [225].
